# 169. Universal Immunization Strategy Against Respiratory Syncytial Virus (RSV) Prevention in Chile with Nirsevimab during the 2024 Winter Season: First Southern Hemisphere Nationwide Effectiveness Data

**DOI:** 10.1093/ofid/ofae631.006

**Published:** 2025-01-29

**Authors:** Juan P Torres, Denis Saure, Miguel O´Ryan, Marcel Goic, Charles Thraves, Natalia Trigo, Gonzalo Diaz, Jorge Pacheco, Javiera Burgos, Patricio Aguilera, Leonardo Basso

**Affiliations:** Universidad de Chile, Santiago, Region Metropolitana, Chile; Universidad de Chile, Santiago, Region Metropolitana, Chile; Universidad de Chile, Santiago, Region Metropolitana, Chile; Universidad de Chile, Santiago, Region Metropolitana, Chile; Universidad de Chile, Santiago, Region Metropolitana, Chile; Universidad de Chile, Santiago, Region Metropolitana, Chile; Universidad de Chile, Santiago, Region Metropolitana, Chile; Ministerio de Salud de Chile, Santiago, Region Metropolitana, Chile; Ministerio de Salud de Chile, Santiago, Region Metropolitana, Chile; Ministerio de Salud de Chile, Santiago, Region Metropolitana, Chile; Universidad de Chile, Santiago, Region Metropolitana, Chile

## Abstract

**Background:**

Nirsevimab for infant RSV prevention was introduced in some Northern Hemisphere countries during 2023. Chile was first to implement a universal countrywide immunization strategy following cost-effectiveness projections in the Southern Hemisphere. This strategy included all newborns born after April 1^st^, 2024, and infants born after October 1^st^, 2023. We report the first national-level impact results of this strategy up to the midwinter period.

**Methods:**

Lower respiratory tract infections (LRTIs) attributed to RSV, causing intensive care (PICU) and overall hospitalizations, is being collected from integrated national databases. For effectiveness, individual hospitalization records are imputed to RSV by best matching ICD-10 codes to the national sentinel program and consolidated hospitalization records. Statistical analyses at different aggregation levels are being conducted to estimate the impact of nirsevimab. Detailed patient-level information is being collected to compare LRTI hospitalizations among those receiving or not nirsevimab using the Cox proportional hazards model. Effectiveness was estimated as (1 – hazard rate) ×100, and 95%CIs were obtained from the Cox model (NCT06511687).

**Results:**

By epidemiological week 23 (mid-June 2024), nirsevimab coverage was 98% and 83% for in-season newborns and the infant catch-up group, with a total of 95,373 doses administered, from a total of 160,000 doses projected up to September 30. Effectiveness was 86% (95%CI 65-95%, 24 cases) and 75% (95%CI 64-83%, 141 cases) against PICU and overall RSV-associated hospitalizations, respectively. Among immunized and non-immunized elegible infants, days of stay were 3.95 (SD 2.46) and 7.87 (SD 6.83) (p=0.31) in the PICU and 3.23 (SD 2.02) vs 4.24 (SD 3.57) (p=0.008) in the pediatric ward, respectively. To date, no RSV deaths have been reported in Chile, compared to 13 cases in 2023. Nirsevimab associated serious adverse events have not been observed.

**Conclusion:**

The nirsevimab-based nationwide immunization program is providing over 75% prevention against RSV associated PICU and overall hospitalizations in Chile, with an optimal safety profile. These results may encourage other similar countries to advance RSV prevention efforts (GRANT ANID PIA AFB230002 ISCI).

**Disclosures:**

**Juan P. Torres, Medical Doctor, PhD**, Pfizer: Honoraria|Sanofi: Honoraria

